# Reasons for treatment discontinuation in the first year after beginning antiretroviral therapy in a cohort of Portuguese HIV-infected patients

**DOI:** 10.1186/1758-2652-13-S4-P13

**Published:** 2010-11-08

**Authors:** C Caldas, P Andrade, C Azevedo, C Piñeiro, R Serrão, J Soares, R Marques, A Sarmento

**Affiliations:** 1 Hospital Sao Joao, Infectious Diseases, Porto, Portugal

## Purpose of study

To evaluate the reasons and risk factors for antiretroviral discontinuation in a cohort of HIV-infected patients in the first year after starting combined antiretroviral therapy (cART) for the first time.

## Methods

A cohort of naïve HIV-infected patients who started cART in the context of a national reimbursement program from January 2007 onwards is being prospectively followed. For each individual, the date and reason of the first discontinuation of any drug in the initial regimen was identified. Changes to same drug formulations were not counted as discontinuations.

## Results

399 patients were enrolled, with a mean follow-up of 82±48 weeks. This study concerns the 248 (62.2%) patients who have a follow-up of ≥1 year. Mean age 42.2±12.3 years (range 78-20), 71.8% were males. 98.8% were HIV-1-infected. HIV was sexually transmitted in 77.8% of the patients, 21.0% were IVDUs; in 1,2% risk was undetermined. HCV/HBV co-infection respectively in 24.6% and 4.4%. At baseline: 28.6% of the patients had AIDS. Mean CD4 cell count 185 cells/mm^3^ (range 2-833). Viral load (VL) >100000 copies/mL in 55.6%. The cART regimen was based in NNRTIs /PIs in respectively 67.3% and 31.9% of the patients.

A total of 109 (44.0%) patients discontinued at least one drug in their cART regimen. The main reasons for discontinuation were: drug related adverse effects (39/35.8%), lack of adherence/lost to follow up (20/18.3%), virological failure (18/16.5%), regimen simplification (17/15.6%), increased CV risk (7/6.4%), pregnancy (4/3.7%). Four patients died. Low median CD4 count (p=0.021) and high median viral load (p=0.075) were found to be associated with virological failure.

## Conclusions

Drug related adverse effects were the main cause of antiretroviral discontinuations in this cohort, with a rate higher than the combined rates for lack of adherence/lost to follow-up and virological failure. Continued attempts to improve the tolerability of cART regimens and patient adherence may help to minimize drug discontinuation rates over the longer term.

